# Healthcare-Associated Infections and Prevention Programs in General Nursing versus Residential Homes—Results of the Point Prevalence Survey in Polish Long-Term Care Facilities

**DOI:** 10.3390/medicina60010137

**Published:** 2024-01-11

**Authors:** Katarzyna Baranowska-Tateno, Agnieszka Micek, Agnieszka Gniadek, Jadwiga Wójkowska-Mach, Anna Różańska

**Affiliations:** 1 Doctoral School of Medical and Health Sciences, Jagiellonian University Medical College, 31-121 Cracow, Poland; k.baranowska-tateno@doctoral.uj.edu.pl; 2 Statistical Laboratory, Institute of Nursing and Midwifery, Faculty of Health Sciences, Jagiellonian University Medical College, 31-126 Cracow, Poland; agnieszka.micek@uj.edu.pl; 3 Faculty of Health Sciences, Jagiellonian University Medical College, 31-501 Cracow, Poland; agnieszka.gniadek@uj.edu.pl; 4 Department of Microbiology, Jagiellonian University Medical College, 31-121 Cracow, Poland; mbmach@cyf-kr.edu.pl

**Keywords:** infection prevention and control, long-term care facilities, healthcare-associated infections, nursing homes, residential homes

## Abstract

*Background and Objectives*: The number of residents of long-term care facilities (LTCFs) is expected to increase. Determining the epidemiological situation in the context of organizational conditions is therefore extremely important for planning the necessary future activities in the field of infection prevention. The aim of this study was to analyze the prevalence rates in Polish nursing vs. residential homes, in the context of the medical and functional burdens of residents and the organizational conditions of both types of units. *Material and Methods*: the data that were analyzed came from a point prevalence survey of infections and antibiotic consumption in LTCFs, conducted in accordance with the HALT-3 protocol in Poland in 2017, between April and June. *Results*: This study included a total of 2313 residents in 24 LTCFs. The most common risk factors for infections in the study population were urinary and fecal incontinence (77.0%), impaired mobility (the patient was in a wheelchair or lying down) (68.7%), and impaired spatial and temporal orientation (52.5%). The median prevalence in nursing homes (NHs) was 3.2% and that in residential homes (RHs) was 0.7%, but without statistical significance. The median for the entire group was 2.6%. A total of 93 healthcare-related infections were detected in 91 residents. The most frequently reported forms of infections were urinary tract infections, lower respiratory tract infections, and skin infections. A statistically significant positive correlation was found only between the percentage of residents with pressure ulcers and other wounds and the incidence of gastrointestinal infections (correlation coefficient = 0.413, *p* < 0.05). Infection prevention and control measures were implemented mainly in nursing homes, and in residential homes, only hand hygiene procedures were commonly available. *Conclusions*: For the two types of LTCFs, the epidemiological situation in terms of nosocomial infections is diverse. Consequently, both types of facilities require different approaches to infection control and prevention and outcomes analysis.

## 1. Introduction

Healthcare-associated infections (HAIs) are a significant, growing problem for healthcare systems around the world. They cause complications, excess deaths, and an increase in the costs of medical care.

The estimated burden of infections with antibiotic-resistant bacteria in the EU and EEA, which are about half of all HAI cases [1], is substantial compared with that of other infectious diseases, larger than the total burden of tuberculosis, HIV, and influenza [2]. According to the analysis of Cassini et al., the population of those aged older than 65 years are characterized with a high burden expressed by DALYs and deaths [2]. Additionally, Poland is one of the European countries with the highest values of these indicators [2]. Point prevalence surveys of healthcare-associated infections (HAIs) and antimicrobial use in the European Union and European Economic Area (EU/EEA) from 2016 to 2017 included 310,755 patients from 1209 acute care hospitals (ACHs) in 28 countries and 117,138 residents from 2221 long-term care facilities (LTCFs) in 23 countries and revealed that 6.5% patients in ACHs and 3.9% residents in LTCFs had at least one HAI [3]. For HAIs in ACHs, there are numerous epidemiological studies, because in most countries, including Poland, the surveillance of infections is obligatory and performed by personnel trained in this area. In the case of long-term care facilities, both infection surveillance and available data on this subject are fragmentary. However, residents of long-term care facilities, due to factors such as old age, auto- and allo-psychical orientation disorders, chronic diseases, urinary and fecal incontinence, the presence of a catheter in the urinary bladder and a vascular catheter, pressure sores, and chronic skin lesions, are exposed to infections [4].

Infection in an elderly patient often initiates a chain of causes leading to hospitalization and death. Also, the housing conditions in long-term care facilities (multi-person rooms, shared dining, activity, and rehabilitation rooms) favor the spread of infections between residents.

Infection surveillance in these units is also a challenge due to the need to implement specific tools, in particular definitions of infection detection that take into account non-specific symptoms of infection [5]. Diagnosing infections in geriatric patients requires, in particular, paying attention to symptoms such as the deterioration of cognitive functions, falls, changes in the nature of chronic symptoms such as cough, secretions remaining in the respiratory tract, and asymptomatic bacteriuria.

Geriatric patients often do not have fever or leukocytosis in the course of infection, and the radiological image during chest examination may also not fully reveal any existing lesions [4,5].

It is expected that due to the aging of the population, the number of residents of long-term care facilities will increase, and therefore an increasing population will be exposed to healthcare-associated infections in these facilities. Importantly, the term “long term care facilities” in Poland and generally in Europe is used for facilities of different types, both in terms of scope of services and patient/resident characteristics. In Poland, there are two main categories of LTCFs: nursing homes (NHs) [6] and residential homes (RHs) [7]. The eligibility criteria vary for admission to NHs or RHs. RH residents are unable to lead an independent life, due to a wide range of reasons, not only medical, but also social, financial, and inadequacy in life [7]. A similar situation concerns the qualification for NHs; in order to be admitted to an NH, a resident must meet certain medical criteria for the severity of health conditions [6]. This is reflected in residents’ general health status and aggravating risk factors for infection.

The availability of medical staff in LTCFs is regulated by law—all NHs must have 24-h nursing care and physician care available on site a minimum of twice a week and on call. RHs, on the other hand, must provide care staff 24/7, whom need not be medical personnel. Medical care is provided by a GP individually selected by the resident at an external facility [6,7].

Infection control in healthcare units is based on professional staff and infection control teams, in particular epidemiological nurses, and is obligatory, with formal regulations similar to hospitals. In residential homes, the implementation of infection control and prevention is not obligatory.

According to data from the Polish Ministry of Health and the Central Statistical Office, at the end of 2017, there were a total of 1190 non-pediatric LTCFs in Poland, including 331 NHs and 859 RHs, with 33,100 and 81,200 beds, respectively [8,9,10]. Poland is the fourth-most populous European country and the number of beds in LTCFs is not sufficient, according to the 2020 report of the Supreme Audit Office [11].

Additionally in Poland, the availability of medical staff, especially doctors and nurses, is much worse than in Europe. According to Eurostat and OECD reports, in 2017 in Poland, the ratio of active doctors was 2.4/1000 patients and that of nurses was 5.1/1000 patients. These were among the lowest results in Europe and much lower than the average for 36 OECD countries, which was 3.5/1000 and 8.8/1000, respectively [12,13,14].

Determining the epidemiological situation in the context of organizational conditions is therefore extremely important for planning the necessary future activities in the field of infection prevention and improving the quality of life of people staying in long-term care facilities.

Since 2008, the ECDC has been conducting surveys on the prevalence of healthcare-associated infections and the use of antimicrobial drugs in long-term care facilities. So far, three editions of the program have been implemented. Poland participated in the last one in 2016–2017, and this study covered 24 facilities, including 12 nursing and 12 residential homes. Aggregate data, without taking into account differences in the type of facility, were included in ECDC publications, and an in-depth analysis, although also based on the aggregate dataset, was also carried out for the use of antibiotics in the context of selected healthcare conditions [15,16].

The aim of this study was to analyze the epidemiology of HAIs expressed by prevalence rates in two main types of long-term cares facilities in Polish nursing vs. residential homes, in the context of the medical and functional burdens of residents and organizational conditions of both types of units.

## 2. Materials and Methods

The data that were analyzed came from a point prevalence survey of infections and antibiotic consumption in long-term care facilities, conducted in accordance with the HALT-3 protocol in Poland in 2017 [15]. All facilities participated in this study on a voluntary basis. This study was conducted in Poland between April and June 2017. The inclusion criterion of the unit was the consent to participate in this study after sending an invitation by the team coordinating it in Poland. The inclusion criterion for residents/patients was the presence in the unit at 8:00 on the day of the survey. Data were collected using standardized unit forms and infection forms, taking into account the occurrence of specific infection risk factors among residents (bladder catheterization, vascular catheterization, pressure sores, condition after surgery in the last 30 days), the residents’ functional status (urinary and/or fecal incontinence, mobility, disturbances of consciousness) Then, after anonymization, data were sent to the coordinating center. In case of an infection and/or use of an antimicrobial drug, the form of infection, its origin (unit, hospital, other), the type of drug, and information about a possible microbiological test were recorded. The unit form additionally included questions about infection control and prevention programs in facilities, including access to medical care, infection control specialists, having procedures for preventing selected forms of infections, and monitoring drug resistance. The study HALT-3 for Poland has received a positive evaluation of the Bioethical Commission of the Jagiellonian University in Krakow (no. 122.6120.64.2017 of 20 June 2017).

To assess the epidemiology of infections in the study group, indicators calculated such as the quotient of the number of infections to the number of residents, in individual units and in total, were used. Due to the low incidence of infections and their skewed distribution across units, the description of the distribution was based on the 75th, 85th, and 95th percentiles (instead of conventional quartiles)—considering that these positional measures better reflect the characteristics of the infection rate.

To assess the statistical significance of the difference in the distribution of individual types of infections between NH and RH units, the non-parametric Mann–Whitney U test was used, and to verify the differences in the proportions of individual infections in all NH and RH units combined, the chi-square test was used, i.e., comparing marginal distributions without taking into account the division into individual units.

Spearman’s rank correlations were used to assess the relationship between the percentage of residents with various burdens and the occurrence of specific types of infections.

## 3. Results

### 3.1. Residents Characteristics and Infections

This study included a total of 2313 residents in 24 LTCFs. The average size of the LTCFs participating in this study was 103.2 beds, the largest facility had 394 beds, and the smallest had 32. The percentage of single rooms averaged 18.8% for all LTCFs (for individual facilities ranging from 0.0% to 87.3%), while the percentage of single rooms with a separate hygiene and sanitary facility was 11.6% (0.0–87.3%).

The most common risk factors for infections in the study population were urinary and fecal incontinence (77.0%), impaired mobility (the patient was in a wheelchair or lying down) (68.7%), and impaired spatial and temporal orientation (52.5%). Differences in the use of invasive medical procedures between individual facilities are noteworthy—in individual centers, the percentage of residents with a urinary catheter ranged from 1% to 77%, and that with an intravascular catheter ranged from 0% to 29%. The percentage of residents with pressure ulcers also varied widely across facilities, from 0% to 24%.

The analysis of residents’ characteristics showed that all aggravating factors, apart from the presence of wounds other than pressure sores, were significantly more common in nursing homes. Characteristics of the study population, including infection risk factors and care burden indicators, are presented in Table 1.

The median prevalence in NHs was 3.2% and that in RHs was 0.7%, but these were not statistically significant differences (Table 2). The median for the entire group was 2.6%.

A total of 93 healthcare-related infections were recorded in all long-term care facilities in 91 residents, of which 88.2% (n = 82) were related to the stay in a given facility and 11.8% (n = 11) to other healthcare facilities. No significant differences were found in the prevalence rates of infections of particular forms, except for respiratory infections classified as other (11 (0.99%) in NHs vs. 2 (0.17%) in RHs, *p* = 0.021, based on the chi-square test) and fungal infections (0 (0.00%) in NHs and 6 (0.51%) in RHs (0.047)) (Table 3).

The most frequently reported forms of infections, both in RHs and NHs, are urinary tract infections, lower respiratory tract infections, and skin infections (Table 4).

A statistically significant positive correlation was found only between the percentage of residents with pressure ulcers and other wounds and the incidence of gastrointestinal infections (correlation coefficient = 0.413, *p* < 0.05). Microbiological testing was performed in only 18.3% of registered infections (n = 17), with a positive result for 17.2% of all infections (n = 16). Pathogens from the *Enterobacterales* family were most frequently isolated—56%, followed by non-fermenting Gram-negative bacilli—16%, Gram-positive cocci—12%, and other—16%.

Antimicrobials were used in 74 LTCF residents (3.19%) during the study period, and the total number of antibiotics prescribed was 82, of which 74 (90.2%) were used for the treatment of infection and 8 (9.2%) were used for prophylaxis. The route of drug administration was oral in 53.7% of cases and parenteral in 43.9%. The dominant groups of antibiotics in the treatment were penicillins (29.9%), other b-lactam antibiotics (25.4%), and fluoroquinolones (19.4%). Prophylactic usage was dominated by drugs classified as “other” (J01X) (87.5%), and in all cases, the reason was to prevent urinary tract infections. In treatment, antimicrobial drugs were most often used for infections of the respiratory system (39.2%) and urinary system (36.5%).

### 3.2. Infection Control and Prevention Programs

In all units where this study was conducted, written procedures regarding HH were available, although 83.3% of NHs and 58.3% of RHs declared the possibility of training in this area (Table 5). All NHs employed ICPs and HAI monitoring was conducted. The possibility of additional consultations with external infection control experts was declared by 58.3% of NHs and half of RHs. Written procedures for the management of patients with urinary catheters were available in the majority of NHs and RHs (91.7% and 58.3%). Access to therapeutic recommendations in the facility regarding respiratory tract infections, urinary tract infections and skin and subcutaneous tissue infections were declared by half of NH patients and 8.3% of RH patients. Monitoring the use of antibiotics (annual report) was declared by 66.7% of NH patients and 25% of RH patients, and the monitoring of microbial resistance was declared by 66.7% and 0%, respectively.

## 4. Discussion

The precise number of LTCFs in Poland is not known—different official sources provide different numbers [9,10,11,15]. Our study included 24 facilities (12 NHs and 12 RHs) with 2281 residents, and there were no broader studies in this field before in Poland.

Residents of nursing homes constituted 48.8% of the surveyed population, and those from residential homes constituted 51.2%. The median number of residents for all the examined facilities was 71.0, that for NHs was 47, and that for RHs was 95.5, and it was a statistically significant difference. Of the European HALT-3 study population, 45.9% were NH residents, 26.5% were mixed LTCFs, and 8.1% were RH residents. The median number of residents in the European institutions surveyed was 44.0 [3]. Compared to the population of European patients included in this study, the Polish population was characterized by a lower percentage of residents over 85 years of age (50% vs. 30.6%) and, at the same time, a worse general condition of patients—there was a higher percentage of patients with impaired mobility (48.8% vs. 68.7%) and with urinary or fecal incontinence (69.3% vs. 77.0%) [3].

The median HAI prevalence for the entire LTCF group of all countries participating in HALT-3 was lower than for Poland and amounted to 2.1% [3]. The average prevalence rate in the present study (2.6%) was almost 50% higher than in Polish LTCFs in the first edition of the HALT program, which was 1.8% [17]. However, our average prevalence rates in both types of LTCFs were lower than those reported by Cotter at al. in Irish LTCFs [18]. In their study, the HAI prevalence was 3.7% with a wide range: 0–22.2%. In an Italian single-center study, Ripabelli et al. reported a prevalence rate of 3.4% [19], and, in a multi-center study conducted by Furmenti et al., it was 3.9% [20] (the protocol and time of study were convergent with ours).

The prevalence rate in our study was almost five times higher in NHs than in RHs. However, this difference was not statistically significant, which may be due to the small size of the study group. The distribution of forms of infections in the study group was consistent with that expected for the geriatric population—infections of the urinary tract, skin, and respiratory system predominated [3,18,19,20,21], although skin infections occurred more frequently than in all countries participating in the HALT-3 program [3]. The analysis of prevalence in relation to infections of various forms revealed differences in NHs vs. RHs only in the case of respiratory tract infections other than pneumonia and colds or flu, and in the case of fungal skin infections. The differences found may be related to the characteristics of infection risk factors affecting the studied population. Residents of nursing homes, as they are less mobile—often bedridden for a long time—are more exposed to respiratory infections [22,23,24], while a greater mobility, and, therefore, self-performed personal hygiene and the use of shared toilets, may increase the incidence of fungal skin infections among nursing home residents [25,26]. The reason for the lack of statistically significant differences, both in terms of total incidence rates and the forms of infections, may be the too small size of the study group (number of infections) or organizational conditions in various types of units. On the other hand, there are more residents in poor health conditions, demanding medical care in nursing homes compared to in residential homes, but only in NHs should infection control programs be implemented in Poland according to formal regulations. This is reflected in the data obtained regarding the presence of written procedures for preventing infections and the implementation of infection control policy, including antibiotic consumption and the drug resistance of microorganisms. Only 52% of facilities in our study declared the functioning of HAI monitoring, which had a lower rate compared to the study of Puto et al. where 81.1% of respondents confirm this [27]. In the Italian multi-center study by Furmenti et al., half of 418 participating settings declared access to professionals trained in infection control [20]. In a study by Puto, 63.9% of respondents from LTCFs declared the implementation of surveillance of multidrug-resistant microorganisms, and in ours, it was 33%. What is interesting in our study is that all facilities declared the presence of written procedures on HH, but only 71% declared training in this area. Hand hygiene is the basic and effective prevention of HAI, but the compliance rates of practice with recommendations depend on continuous and real, powerful training [28,29]. This observation on the implementation of basic procedures for prevention of the transmission of resistant microorganisms is alarming. Chen et al. in their review study report that the prevalence rate of carbapenem-resistant *Enterobacterales* (CRE) in LTCFs was significantly higher than that in acute care settings and communities [30]. They claim LTCFs as a vital reservoir for those strains and, among others, indicate fecal incontinence, indwelling devices, comorbidities, dementia, and dependent functional status as risk factors for CRE acquisition. In our study, 56% of strains isolated in HAIs were from the *Enterobacterales* family. The effective implementation of ICP procedures is also crucial for the prevention of viral infections of the respiratory tract, including influenza [31], but our study suggests that substantial improvements should be made in Polish settings in this area.

However, microbiological tests were performed for 18.47% of registered infections and this is a result close to the European average tested, which was 19.2% [3], but less than in the study of Furmenti, with 26.4% HAIs with a microbiological diagnosis [20]. The lack of sufficient microbiological diagnostics is a common problem in healthcare settings in LTCFs [32], as well as the use of antibiotics that is probably not entirely consistent with recommendations [33,34,35]. Due to diagnostic difficulties typical of the geriatric patient population, some overdiagnosis and, consequently, overuse of antibiotics can be expected. This is a vital problem most common in elderly patients and consequently in LTCF urinary tract infections [36,37], but also other infections associated with invasive devices and biofilm formation, most often encountered in LTCFs [38]

### Strengths and Limitations of This Study

Our study is a unique source of information about HAIs in Polish LTCFs. However, it has several limitations. Among other things, the number of LTCFs is small, although much larger than in the first edition of the HALT project. The second limitation is the methodology itself—point prevalence studies are, by nature, preliminary and allow for the general estimation of epidemiology, and they do not reflect the situation throughout the year, although infections are characterized by seasonal variability. The relatively small study group does not allow the capture of all correlations and their possible significance. Additionally, this study was conducted in accordance with an established protocol that took into account the most important variables that could be recorded. The test was performed before the COVID-19 pandemic, so this infection was not included. Another edition of this point prevalence survey is currently taking place, based on a modified form, which will allow for the assessment of the post-pandemic situation.

## 5. Conclusions

For the two main types of long-term care facilities operating in the Polish system, the epidemiological situation in terms of nosocomial infections is diverse. The reasons should be attributed to the varied characteristics of residents/patients. Consequently, both types of facilities require different approaches to infection control and prevention and outcomes analysis.

Regardless of the type of entity, the most common are infections of the urinary tract, skin, and subcutaneous tissue, and infections of the respiratory system, which can be significantly reduced by improving hygiene and care procedures, including urinary bladder catheterization.

The weakest points of infection control policy are the ongoing monitoring of healthcare-associated infections, antibiotic use, and antibiotic resistance. Moreover, guidelines for antibiotic therapy in the most common infections are poorly available.

All these observations indicate the urgent need of the implementation a common, coordinated on the national level, system of infection prevention and control in long-term care facilities in Poland.

## Figures and Tables

**Table 1:** Residents’ characteristics.

Parameter	All LTCF		NH		RH		NH vs. RH
Number of Facilities	24		12		12		
Total Number of Residents	2281		1114		1167		
Descriptive Parameters According to Facilities	Q2 (Q1–Q3)	MIN–MAX	Q2 (Q1–Q3)	MIN–MAX	Q2 (Q1–Q3)	MIN–MAX	*p* ^#^
Residents, n	71.0	19–389	47.0 (40.8–89.0)	33–389	95.5 (61.8–114.8)	19–229	0.453
	(40.8–114.8)						
Men, %	32.7 (26.0–42.7)	0–56	29.4 (16.6–31.6)	0–40	44.2 (36.2–52.0)	19–56	0.002
Residents with urinary catheter, %	8.4 (3.5–21.2)	1–77	19.2 (11.9–47.7)	1–77	4.3 (1.8–7.2)	1–21	0.002
Residents with vascular catheters %	0.0 (0.0–6.6)	0–29	7.1 (3.2–11.7)	0–29	0.0 (0.0–0.0)	0–1	<0.001
Residents with pressure sore, %	7.7 (4.6–11.5)	0–24	11.7 (7.7–14.7)	2–24	5.4 (2.7–7.6)	0–10	0.005
Residents with other wounds, %	3.6 (1.9–8.5)	0–24	4.3 (2.4–9.8)	0–24	2.5 (1.5–5.4)	0–11	0.284
Place/time-disoriented residents, %	52.5 (42.1–62.6)	7–92	62.9 (54.0–68.9)	40–92	45.2 (34.3–52.0)	7–62	0.004
Bedridden or on-wheel residents, %	68.7 (44.8–85.7)	33–100	86.6 (77.5–96.1)	69–100	44.5 (40.7–47.4)	33–68	<0.001
Residents with incontinence, urine, or stool, %	77.0 (58.5–88.4)	44–100	86.5 (81.8–94.9)	69–100	57.3 (49.1–63.6)	44–95	<0.001

Q2—median, Q1—lower quartile, Q3—upper quartile, NH—nursing homes, RH—residential homes, LTCF—Long-Term Care Facilities; ^#^ *p* comparing the distributions of individual variables between NH and RH units calculated on the basis of a non-parametric U Mann–Whitney test.

**Table 2:** Prevalence of healthcare-associated infections in LTCF.

Prevalence—All HAI, %						
All LTCF		NH		RH		NH vs. RH
Q2 (Q1–Q3)	MIN–MAX	Q2 (Q1–Q3)	MIN–MAX	Q2 (Q1–Q3)	MIN–MAX	*p* ^#^
2.6 (0.0–5.4)	0–19	3.2 (2.5–4.6)	0–17	0.7 (0.0–5.4)	0–19	0.251

Q2—median, Q1—lower quartile, Q3—upper quartile, NH—nursing homes, RH—residential homes, LTCF—Long-Term Care Facilities. ^#^ *p* comparing the distributions of individual variables between NH and RH units calculated on the basis of a non-parametric U Manna Whitney test.

**Table 3:** Prevalence of healthcare-associated infections in study group, according to infection type and type of setting.

Infection	All LTCF			NH			RH			NH vs. RH	
	n (%) ^@^	P85 (P75–P95)	MIN–MAX	n (%) ^@^	P85 (P75–P95)	MIN–MAX	n (%) ^@^	P85 (P75–P95)	MIN–MAX	*p* ^#^	*p* ^&^
Urinary tract infection	27 (1.18%)	3.2 (1.7–6.0)	0–9	14 (1.26%)	2.2 (1.4–5.8)	0–9	13 (1.11%)	3.8 (2.7–5.7)	0–6	0.949	0.903
Confirmed	7 (0.31%)	0.0 (0.0–3.9)	0–5	1 (0.09%)	0.0 (0.0–0.6)	0–1	6 (0.51%)	1.5 (0.0–4.8)	0–5	0.514	0.146
Not confirmed	20 (0.88%)	2.2 (1.1–3.4)	0–9	13 (1.17%)	2.1 (1.1–5.8)	0–9	7 (0.60%)	2.0 (1.1–2.7)	0–3	0.918	0.220
Respiratory tract infections	21 (0.92%)	2.4 (1.4–3.3)	0–4	14 (1.26%)	2.2 (1.5–3.4)	0–4	7 (0.60%)	1.9 (1.0–3.2)	0–3	0.765	0.155
Cold and throat infections	3 (0.13%)	0.0 (0.0–0.7)	0–2	0 (0.00%)	0.0 (0.0–0.0)	0–0	3 (0.26%)	0.3 (0.0–1.3)	0–2	0.166	0.265
Pneumonia	5 (0.22%)	0.3 (0.0–2.6)	0–3	3 (0.27%)	0.2 (0.0–1.6)	0–3	2 (0.17%)	0.5 (0.0–2.1)	0–3	0.965	0.959
Other respiratory tract infections	13 (0.57%)	0.8 (0.0–1.8)	0–4	11 (0.99%)	1.5 (0.3–2.9)	0–4	2 (0.17%)	0.0 (0.0–0.8)	0–2	0.286	0.021
Skin infections	26 (1.14%)	1.4 (0.8–8.1)	0–9	9 (0.81%)	1.4 (0.9–4.6)	0–9	17 (1.46%)	2.4 (0.2–6.8)	0–9	0.407	0.207
Skin and subcutaneous tissue, wounds	20 (0.88%)	1.4 (0.8–4.9)	0–9	9 (0.81%)	1.4 (0.9–4.6)	0–9	11 (0.94%)	1.8 (0.2–4.2)	0–5	0.370	0.904
Fungal infections	6 (0.26%)	0.0 (0.0–0.0)	0–5	0 (0.00%)	0.0 (0.0–0.0)	0–0	6 (0.51%)	0.0 (0.0–2.4)	0–5	0.359	0.047
Surgical site infections	2 (0.09%)	0.0 (0.0–0.7)	0–1	0 (0.00%)	0.0 (0.0–0.0)	0–0	2 (0.17%)	0.3 (0.0–0.9)	0–1	0.166	0.500
Eyes, ears, nose, oral cavity infections	8 (0.35%)	0.0 (0.0–1.7)	0–4	1 (0.09%)	0.0 (0.0–0.6)	0–1	7 (0.60%)	0.6 (0.0–2.9)	0–4	0.514	0.088
Conjuctivitis	5 (0.22%)	0.0 (0.0–0.7)	0–3	0 (0.00%)	0.0 (0.0–0.0)	0–0	5 (0.43%)	0.3 (0.0–2.0)	0–3	0.166	0.082
Ears	3 (0.13%)	0.0 (0.0–0.9)	0–1	1 (0.09%)	0.0 (0.0–0.6)	0–1	2 (0.17%)	0.3 (0.0–0.9)	0–1	0.651	1.000
Gastrointestinal infections	8 (0.35%)	1.4 (0.9–2.7)	0–3	6 (0.54%)	2.5 (1.7–2.9)	0–3	2 (0.17%)	0.3 (0.0–0.9)	0–1	0.106	0.259
Stomach and bowel	4 (0.18%)	0.0 (0.0–0.9)	0–1	2 (0.18%)	0.0 (0.0–0.6)	0–1	2 (0.17%)	0.3 (0.0–0.9)	0–1	0.651	1.000
*C. difficile*	2 (0.09%)	0.0 (0.0–2.3)	0–3	2 (0.18%)	0.9 (0.0–2.9)	0–3	0 (0.00%)	0.0 (0.0–0.0)	0–0	0.166	0.459
Bloodstream	1 (0.04%)	0.0 (0.0–0.0)	0–1	1 (0.09%)	0.0 (0.0–0.6)	0–1	0 (0.00%)	0.0 (0.0–0.0)	0–0	0.359	0.981
Other	1 (0.04%)	0.0 (0.0–0.0)	0–2	1 (0.09%)	0.0 (0.0–1.1)	0–2	0 (0.00%)	0.0 (0.0–0.0)	0–0	0.359	0.981

P75–75, P85–85, P95–95, percentiles of distribution; NH—nursing homes; RH—residential homes; LTCF—Long-Term Care Facilities; ^@^ distribution calculated as number of infections per number of residents; ^#^ *p* comparing the distributions of individual variables between NH and RH units calculated on the basis of a non-parametric U Mann–Whitney test; ^&^ *p* verifying differences in distribution of types of infections in all NH and RH in total according to chi-square test.

**Table 4:** Distribution according to types of healthcare-associated infections in nursing vs. residential homes.

Infection	All LTCF	NH	RH
	n (%) ^^^	n (%) ^^^	n (%) ^^^
Urinary tract infection	27 (28.72%)	14 (30.43%)	13 (27.08%)
Confirmed	7 (7.45%)	1 (2.17%)	6 (12.50%)
Not confirmed	20 (21.28%)	13 (28.26%)	7 (14.58%)
Respiratory tract infections	21 (22.34%)	14 (30.43%)	7 (14.58%)
Cold and throat infections	3 (3.19%)	0 (0.00%)	3 (6.25%)
Pneumonia	5 (5.32%)	3 (6.52%)	2 (4.17%)
Other respiratory tract infections	13 (13.83%)	11 (23.91%)	2 (4.17%)
Skin infections	26 (27.66%)	9 (19.57%)	17 (35.42%)
Skin and subcutaneous tissue, wounds	20 (21.28%)	9 (19.57%)	11 (22.92%)
Fungal infections	6 (6.38%)	0 (0.00%)	6 (12.50%)
Surgical site infections	2 (2.13%)	0 (0.00%)	2 (4.17%)
Eyes, ears, nose, oral cavity infections	8 (8.51%)	1 (2.17%)	7 (14.58%)
Conjuctivitis	5 (5.32%)	0 (0.00%)	5 (10.42%)
Ears	3 (3.19%)	1 (2.17%)	2 (4.17%)
Gastrointestinal infections	8 (8.51%)	6 (13.04%)	2 (4.17%)
Stomach and bowel	4 (4.26%)	2 (4.35%)	2 (4.17%)
*C. difficile*	2 (2.13%)	2 (4.35%)	0 (0.00%)
Bloodstream	1 (1.06%)	1 (2.17%)	0 (0.00%)
Other	1 (1.06%)	1 (2.17%)	0 (0.00%)

^^^ Distribution of infection cases calculated as a number of infections of given type per all infections in a given type of facility.

**Table 5:** Infection prevention and control, antibiotics stewardship.

Type of Process Measures in the Facility	LTCF (%)	NH (%)	RH (%)
Infection prevention and control (IPC)			
Presence of person(s) trained in IPC	68.0	100.0	33.3
Presence of infection control team	52.0	91.7	8.3
Access to consultation with external experts in infection control	52.0	58.3	50.0
Training on hand hygiene for personel in the facility	72.0	83.3	58.3
Presence of written procedures and therapeutic recommendations			
Access to written procedure concerning work with patients infected or colonized with MDRO	64.0	83.3	50.0
Access to written procedures on hand hygiene	100.0	100.0	100.0
Access to written procedure concerning work with patients with urinary catheters	76.0	91.7	58.3
Access in facility to recommendations on treatment of respiratory tract infections	28.0	50.0	8.3
Access in facility to recommendations on treatment of urinary tract infections	28.0	50.0	8.3
Access in facility to recommendations on treatment of skin and soft tissues infections	28.0	50.0	8.3
Surveillance			
Healthcare-associated infections surveillance	52.0	100.0	0
Antibiotic consumption surveillance (yearly report)	44.0	66.7	25.0
Antimicrobial resistance surveillance	32.0	66.7	0

## Data Availability

The datasets used and/or analyzed during the current study are available from the corresponding author upon reasonable request.
